# Application of SHAP for Explainable Machine Learning on Age-Based Subgrouping Mammography Questionnaire Data for Positive Mammography Prediction and Risk Factor Identification

**DOI:** 10.3390/healthcare11142000

**Published:** 2023-07-11

**Authors:** Jeffrey Sun, Cheuk-Kay Sun, Yun-Xuan Tang, Tzu-Chi Liu, Chi-Jie Lu

**Affiliations:** 1 Department of Acute Medicine, West Middlesex University Hospital, London TW7 6AF, UK; 2 School of Medicine, Imperial College London, London SW7 2BX, UK; 3 Division of Hepatology and Gastroenterology, Department of Internal Medicine, Shin Kong Wu Ho-Su Memorial Hospital, Taipei 11101, Taiwan; 4 Graduate Institute of Business Administration, Fu Jen Catholic University, New Taipei City 24205, Taiwan; 5 School of Medicine, Fu Jen Catholic University, New Taipei City 24205, Taiwan; 6 School of Medicine, Taipei Medical University, Taipei 11031, Taiwan; 7 Department of Radiology, Shin Kong Wu Ho-Su Memorial Hospital, Taipei 11101, Taiwan; 8 Department of Medical Imaging and Radiological Technology, Yuanpei University of Medical Technology, Hsinchu 30015, Taiwan; 9 Artificial Intelligence Development Center, Fu Jen Catholic University, New Taipei City 24205, Taiwan; 10 Department of Information Management, Fu Jen Catholic University, New Taipei City 24205, Taiwan

**Keywords:** mammography, breast cancer, explainable machine learning, SHAP value

## Abstract

Mammography is considered the gold standard for breast cancer screening. Multiple risk factors that affect breast cancer development have been identified; however, there is an ongoing debate regarding the significance of these factors. Machine learning (ML) models and Shapley Additive Explanation (SHAP) methodology can rank risk factors and provide explanatory model results. This study used ML algorithms with SHAP to analyze the risk factors between two different age groups and evaluate the impact of each factor in predicting positive mammography. The ML model was built using data from the risk factor questionnaires of women participating in a breast cancer screening program from 2017 to 2021. Three ML models, least absolute shrinkage and selection operator (lasso) logistic regression, extreme gradient boosting (XGBoost), and random forest (RF), were applied. RF generated the best performance. The SHAP values were then applied to the RF model for further analysis. The model identified age at menarche, education level, parity, breast self-examination, and BMI as the top five significant risk factors affecting mammography outcomes. The differences between age groups ranked by reproductive lifespan and BMI were higher in the younger and older age groups, respectively. The use of SHAP frameworks allows us to understand the relationships between risk factors and generate individualized risk factor rankings. This study provides avenues for further research and individualized medicine.

## 1. Introduction

Breast cancer is currently the most diagnosed non-skin cancer in women and ranks 5th among cancer-related deaths worldwide according to GLOBOCAN 2020 data [1]. With an estimated more than 2 million cases each year, the incidence of breast cancer has increased rapidly in recent decades owing to enhanced cancer detection and registration, in addition to the ever-evolving risk factor profile of the population. Globally, Asia had the highest disease burden in 2020, accounting for 45.4% of all new breast cancer cases [2]. In Taiwan, the age-standardized breast cancer incidence rate increased from 60.35 to 128.20 per 100,000 from 1997 to 2016 [3,4]. In response to the increasing disease burden, Taiwan introduced a nationwide biennial mammography screening program in 2004 for women aged 50–69. In 2009, the program was further expanded to encompass women aged 45–69 years and, in 2010, to include women aged 40–44 years who were deemed high risk [5]. Other recent international guidelines encompass a similar age range, with European guidelines recommending annual screening from 45–69 years for asymptomatic women with average risk and American Cancer Society guidelines strongly recommending annual screening for women aged 45–54 years [6,7,8].

Mammography is currently the gold standard for breast screening, with research showing that mammography may be able to detect breast cancer as early as four years prior to cancer being clinically evident [9]. Ongoing advancements in mammography technology aim to provide earlier detection of breast pathology, better visualization of the disease extent, and an accurate assessment of treatment response. Similarly, advances in breast cancer therapy have led to a significant reduction in mortality and increased survivability; however, early diagnosis remains the most crucial factor in contributing toward good prognostic outcomes [10].

Multiple risk factors can influence an individual’s predisposition to breast cancer, given the heterogeneous nature of the disease. There are significant risk factors for breast cancer, divided into modifiable factors such as alcohol intake, smoking, parity, obesity, and hormonal replacement therapy, and non-modifiable factors such as age, sex, family history, menstruation history, and genetic predisposition [11]. With the development of the Gail model in 1989, previous attempts have been made to stratify the relative risk of developing breast cancer in the general population based on the presence of risk factors, which calculates an individual’s combined risk of developing invasive breast cancer [12]. This model accounts for age, ethnicity, menstrual history, parity, family history, and past medical history to provide five-year and lifetime risks of invasive breast cancer. However, it recognizes that the accuracy for ethnic subgroups needs to be further validated. The model also fails to consider many modifiable risk factors that significantly influence an individual’s estrogen and androgen exposure [13].

Our previous study [14] concluded that age was the most impactful factor in predicting positive mammography findings. To further scrutinize the effects of other risk factors on mammography outcomes, this study stratified participants into two groups: women aged 45–49 and 50–54. This range effectively covers the ages with the highest breast cancer incidence rates in Asia [4,15] and allow us to observe any differences that may arise when comparing younger and older age groups. 

It is difficult to rank the relative significance of each risk factor using conventional research methods. In response, recent technological advancements in machine learning have allowed its incorporation into clinical decision making to facilitate medical image interpretation, outcome prediction, and treatment selection. Machine learning (ML) is a subfield of artificial intelligence that uses statistical models to analyze large datasets and interpret the complex interactions between multiple variables and patient outcomes through its automated ability to learn and enhance its analysis from experiences [16]. However, the increased complexity of ML models has created a ‘black box’ phenomenon, whereby the final interpretations through ML methods are incomprehensible and difficult to explain [17]. The enigmatic nature and lack of transparency of ML limit its promising prospects and act as undeniable obstacles to its integration into medical decision making. The need for a comprehensive approach to facilitate the interpretation of ML models has led to the introduction of the Shapley Additive Explanation (SHAP) methodology, which enables the identification and prioritization of the impact of each feature in any ML model [18,19]. This framework provides explainable insights into the ML ‘black box,’ allowing for the rationalization and interpretation of the ML-derived outcomes [20]. 

This study used three ML models, namely, least absolute shrinkage and selection operator logistic regression (lasso) [21,22], extreme gradient boosting (XGBoost) [23], and random forest (RF) [24], in conjunction with SHAP to analyze and stratify the breast cancer risk factors between two different age groups and evaluate the impact of each factor in predicting positive mammography outcomes.

## 2. Methods

### 2.1. Study Design and Protocol

For this retrospective single-center study, relevant data were extracted from risk factor questionnaires completed by women who participated in the national breast cancer screening program between 2017 and 2021 at Shin-Kong Wu Ho-Su Memorial Hospital, Taipei. The risk factor questionnaires were standardized and issued by the Ministry of Health and Welfare in Taiwan. 

**Figure 1:**
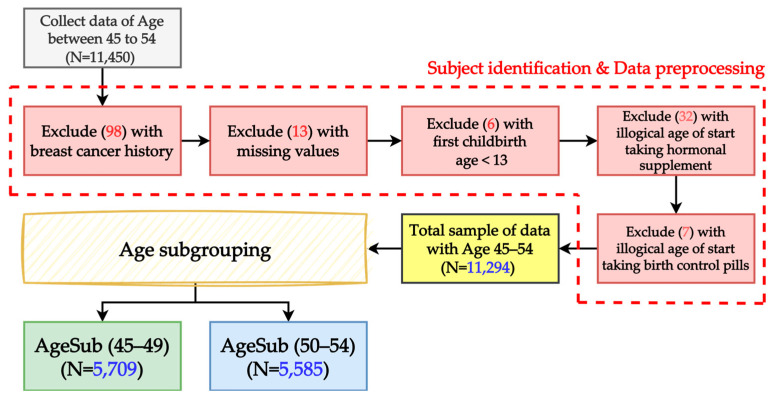
Data preprocessing.

Extensive measures were taken to ensure data quality. This study examined the risk factors for women aged 45–54 years. Participants not in this age group were excluded. Other exclusion criteria included participants with a previous history of breast cancer, as well as questionnaires with missing, inconsistent, or illogical data (Figure 1). The participants were divided into two subgroups for analysis: 45–49 years and 50–54 years. The study protocol and procedures were reviewed and approved by the Research Ethics Review Committee of Shin-Kong Wu Ho-Su Memorial Hospital, which waived the requirement for informed consent from the participants before routine examinations (No. 20220906R).

### 2.2. Variable Definitions and Descriptive Statistics 

The mammography result (Y) was separated into binary outcomes, positive and negative, with reference to Breast Imaging Reporting and Data Systems (BI-RADS) classification [25]. Positive mammography findings were defined as films that were probably benign (BI-RADS 3), suspicious (BI-RADS 4), highly suggestive of malignancy (BI-RADS 5), biopsy-proven (BI-RADS 6), or incomplete imaging (BI-RADS 0). Negative mammography findings were defined as films showing negative or benign findings (BI-RADS 1 and BI-RADS 2, respectively).

A total of 16 separate risk factors were identified as potential predictors of mammography outcomes, all of which were stratified into categories for analysis. Table 1 presents the demographic characteristics of the participants (5 factors in total), whereas Table 2 presents the clinical characteristics of the participants (11 factors in total). Accounting for demographic limitations and data collection purposes, reproductive lifespan was determined from the onset of menarche until menopause in postmenopausal women or until the date of mammography for those who were premenopausal.

### 2.3. Machine Learning Methods

To demonstrate and utilize a plug-in such as SHAP to explain the outcome of an ML model, an ML model that has reasonable performance for the data used in this study should be built first. Thus, three commonly used ML models were used in this study: lasso, XGBoost, and RF. Lasso is a logistic regression (LGR) that adds L1 regularization (least absolute shrinkage and selection operator). LGR is an extension of linear regression (LR) that can handle binary classification problems by converting the outcomes from LR to a value space between zero and one using a logit function (the natural logarithm of an odds ratio) [21]. L1 regularization is a common technique used in regression methods to achieve more accurate predictions using shrinkage. Shrinkage involves moving data values toward a central point, such as the mean. Through shrinkage, the variables that contributed the least to the outcome were dropped [22]. XGBoost is a popular and effective ML method based on the gradient-boosting framework and is combined with other techniques to make it more effective. The main concept of XGBoost is to combine several weak models into a strong model, this is achieved through a straightforward process that involves iteratively adding new models to XGBoost and adjusting the weights of the samples based on the errors made by the previous model until the most optimized performance is reached. In other words, XGBoost self-optimizes when constructing [23]. RF is a popular ensemble-based decision tree (DT) ML method. During modeling, RF first builds multiple uncorrelated forests of DTs from an ensemble using a bagging approach, where each DT is built with randomly selected features and samples from the input data. Then, RF takes the approach of majority voting to output the final prediction [24].

### 2.4. Shapley Additive Explanations (SHAP)

The explainability of an ML method may be limited owing to its mechanism; thus, methods designed to improve explainability have been created and explored recently. Developed by Lundberg and Lee, SHAP was designed to explain the predicted outcomes of an ML model [18]. SHAP extends and utilizes the concept of Shapley values from cooperative game theory and more clinical studies have begun to explore its usage recently [26,27,28]. The basic concept of SHAP is to assign a contribution value to each feature of a predicted outcome. The concept that SHAP uses to calculate contribution values is straightforward. It is calculated by comparing the prediction made with the feature present to the prediction made without the feature present, and the difference between these two predictions represents the contribution of that feature. The contribution of each feature to a predicted outcome may vary (it can affect the outcome positively or negatively) [18]. In addition, SHAP considers all possible combinations of features when calculating the contribution of each feature to the prediction. Overall, in this study, the information provided by the SHAP method could help gain better insight into how each feature in an ML model affects the predicted outcome. 

### 2.5. Proposed Scheme 

**Figure 2:**
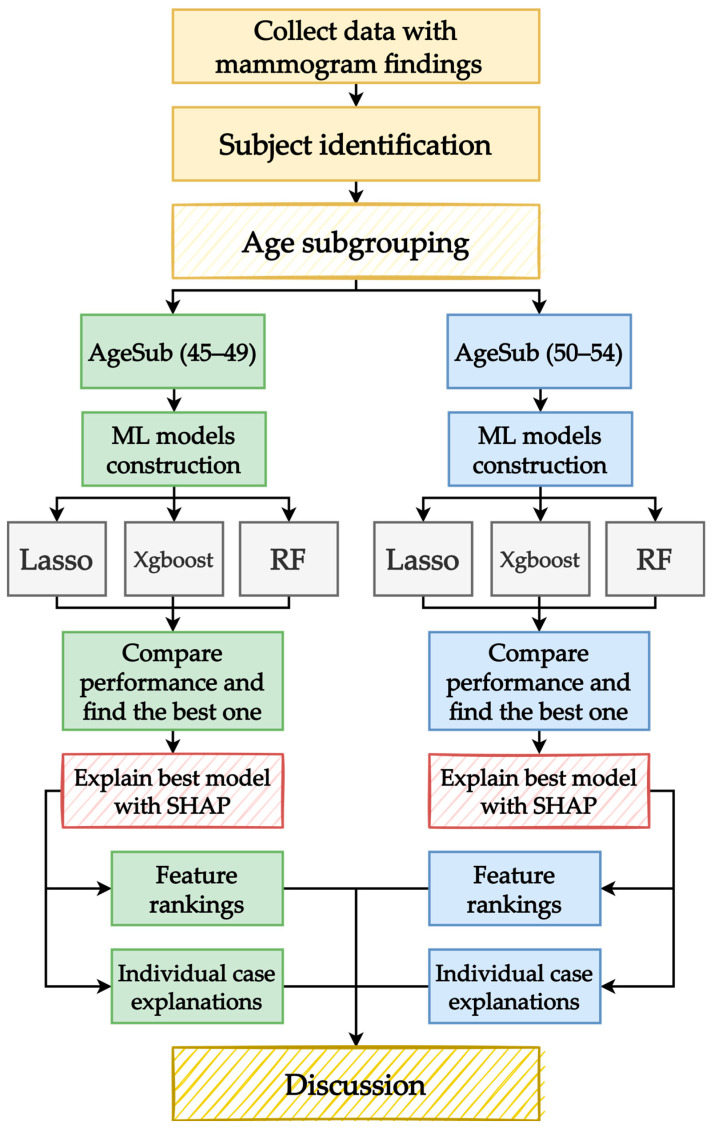
Proposed scheme.

The aim of this study was to explore important features that may affect subjects with the potential for positive breast cancer in different age subgroups. Figure 2 illustrates the proposed analysis scheme. In the proposed scheme, three ML models are built with data from different age subgroups, and outcomes from the best ML model in terms of performance are explained using the SHAP method.

In the proposed scheme, data from the mammogram findings are first collected, and data preprocessing is conducted to exclude subjects that do not satisfy the protocols of the study. After the data were cleaned, they were further divided into two age subgroups, namely, between 45 and 49 years (AgeSub (45–49)) and between 50 and 54 years (AgeSub (50–54)). The three ML models (RF, XGBoost, lasso) were then utilized for modeling both subgroups of data. For each ML model, during the construction process, the data were divided into portions for training (80%) and testing (20%). Because ML methods have hyperparameters that must be tuned, the training portion was further split into portions for training and validation. This study takes the fivefold cross-validation (5f-CV) approach when tuning the hyperparameters. The concept of 5f-CV is straightforward: the training data are randomly split into five folds, and each fold is utilized for validation once. 

After finding the best hyperparameters for each ML model, the testing data were used for performance evaluation to find the best-performing one for each age subgroup. The metrics evaluated were sensitivity, specificity, and area under the receiver operating characteristic curve (AUC). Next, the SHAP method was used to explain the predicted outcomes from the best ML models for each age subgroup. Using SHAP, the overall feature importance rankings of each age subgroup and explanations for individual cases were extracted. Finally, discussions were formed based on the extracted information. 

The experiment was implemented in Python (version 3.8.8) [29] and Jupyter Notebook (version 6.3.0) [30]. Lasso and RF were constructed using the Scikit-learn package API (version 0.24.2) [31,32]; XGBoost was constructed with the XGBoost package (version 1.3.3) [23]; cross-validation and hyper-parameter tuning were conducted with the Scikit-learn API [31,32]. 

## 3. Results

### 3.1. Machine Learning Model Result

Following the scheme mentioned previously, the ML modeling results for the different age subgroups are presented in Table 3. In Table 3, the ML results of AgeSub (45–49) can be seen. The AUC of RF (AUC = 61.62) was more reasonable than those of lasso and XGBoost. Additionally, RF performed more reasonably in terms of sensitivity (54.98) and specificity (64.42) than lasso and XGBoost. Using a concept similar to that when viewing AgeSub (45–49), AgeSub (50–54) can also been seen in the table. As shown in the table, in AgeSub (50–54), RF performed better in terms of AUC (61.78) and sensitivity (66.67) than lasso and XGBoost. In summary, according to the experimental results from this study, RF performs reasonably well in both age subgroups, and a table for comparing the RF performance in each age subgroup is presented in Table 4.

As shown in Table 4, the AUC of the RF in both age subgroups was similar; however, the RF performed differently in terms of sensitivity and specificity. The sensitivity of RF in AgeSub (50–54) was higher than that in AgeSub (45–49), which indicates that RF captures positive cases more effectively in individuals between 50 and 54 years of age, whereas RF captures negative cases well between 45 and 49 years of age. Next, the important features and predicted outcomes of RF in each age subgroup were explained using the SHAP method. 

### 3.2. Average Impact on RF Model Output Magnitude

**Figure 3:**
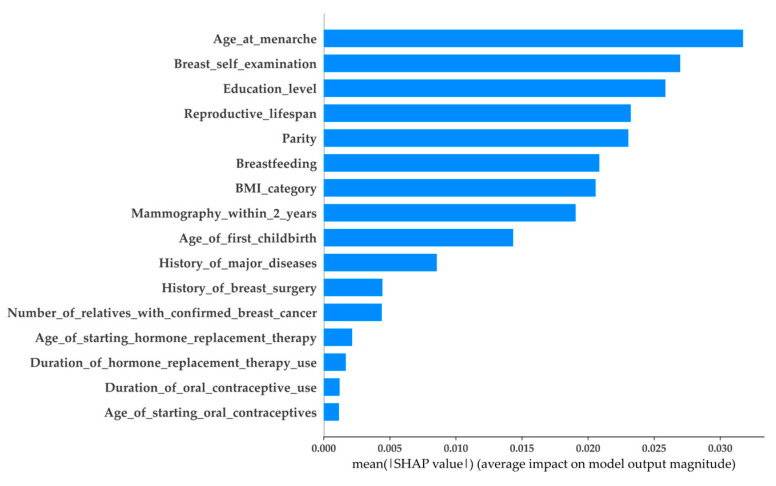
Average impact of SHAP value on RF with AgeSub (45–49).

**Figure 4:**
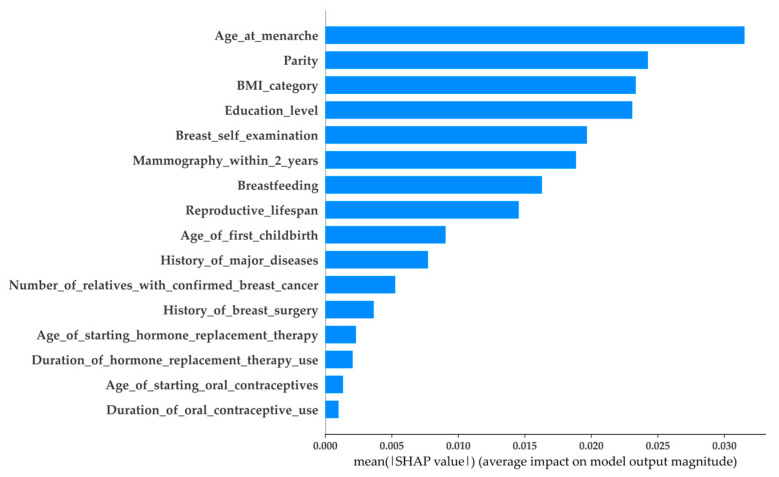
Average impact of SHAP value on RF with AgeSub (50–54).

As mentioned in the previous section, each case may be affected by features with positive or negative SHAP values. These SHAP values indicate the impact of the features on each output (the predicted outcome from the model). To understand the overall impact of each feature on the outputs, the average impact value (AIV) can be utilized. Calculating AIV is straightforward: first, the SHAP values of each feature are transformed to absolute SHAP values; then, the AIV of each feature is the mean of its absolute SHAP values. Finally, the importance of the features can be ranked according to their corresponding AIV, of which the top ranking feature is that with the highest AIV. Figure 3 and Figure 4 present the AIV on the RF with AgeSub (45–49) and AgeSub (50–54), where the y-axis represents the features sorted according to the importance rankings from top to bottom. As shown in the figures, the RF with different age subgroups had features that were ranked differently. For example, in AgeSub (45–49), the top three ranking features were age at menarche, breast self-examination, and education level, whereas age at menarche, parity, and BMI category were the top three ranking features in AgeSub (50–54).

**Figure 5:**
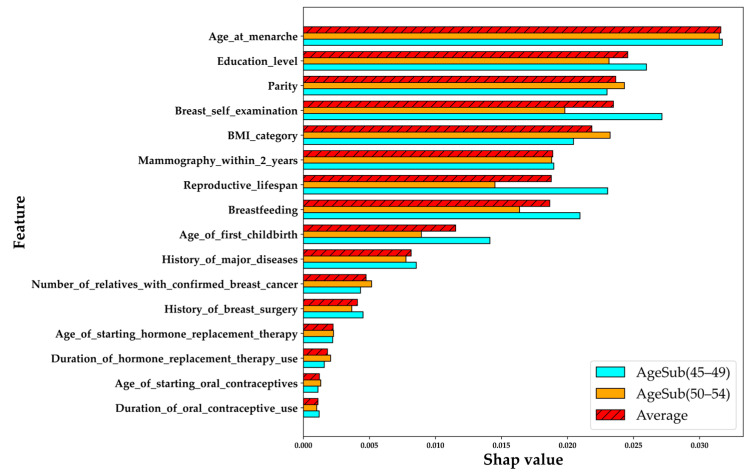
Overall average impact comparison of SHAP value of RF with different age subgroups.

Figure 5 shows the overall average impact of each feature on the outcomes. Three legends can be found in the figure, namely, AgeSub (45–49) (marked with the color cyan); AgeSub (50–54) (marked with the color orange); and Average (marked with the color red and diagonal black lines). Additionally, the legend average was calculated by averaging the AIVs of the features in AgeSub (45–49) and AgeSub (50–54). The y-axis presents the features sorted according to Average, and the one with the highest average AIV is the top-ranking feature. Overall, based on the information shown in Figure 5, age at menarche, education level, parity, breast self-examination, and BMI were the top five features.

### 3.3. Demonstration of Explaining Individual Cases with SHAP Value

**Figure 6:**
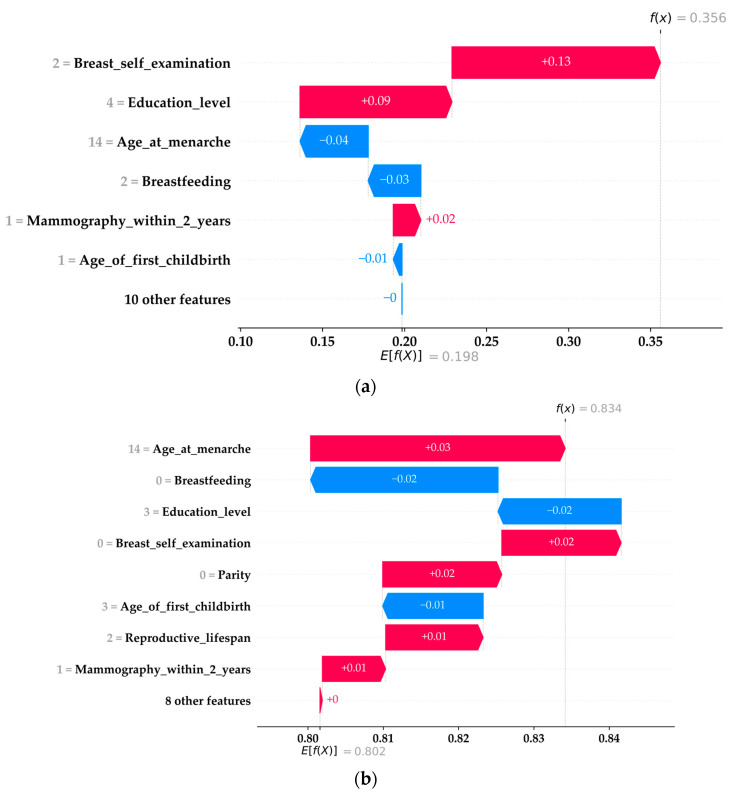

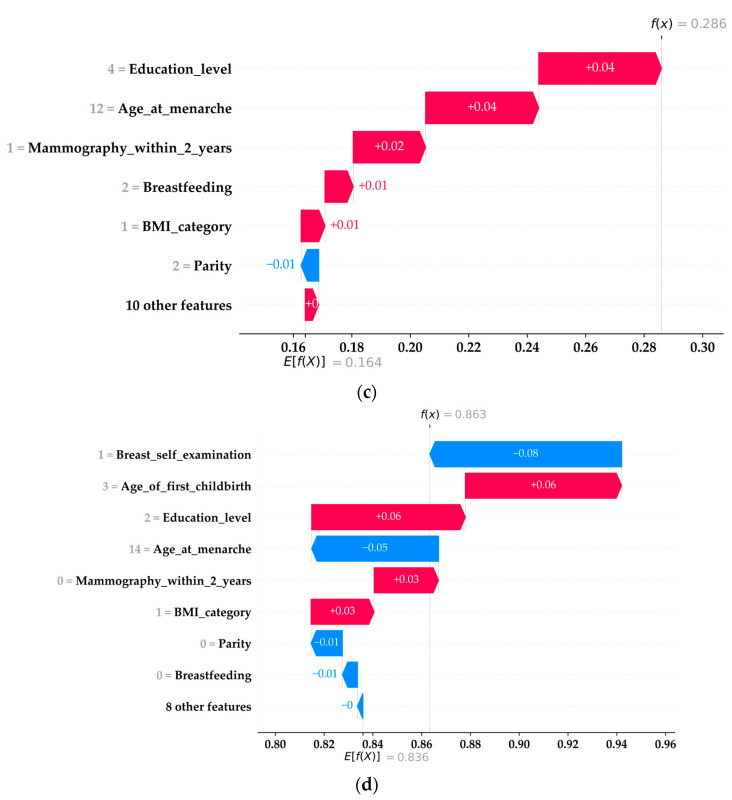
(**a**) SHAP value of individual case of RF in AgeSub (45–49): positive case. (**b**) SHAP value of individual case of RF in AgeSub (45–49): negative case. (**c**) SHAP value of individual case of RF in AgeSub (50–54): positive case. (**d**) SHAP value of individual case of RF in AgeSub (50–54): negative case.

In addition to ranking the importance of the features, the SHAP method can explain how each feature impacts individual outcomes in the RF model. Figure 6a,b show the demonstrations of SHAP explaining a positive case (Figure 6a) and a negative case (Figure 6b) in Ag3222eSub (45–49), while Figure 6c (positive case) and Figure 6d (negative case) are demonstrations of explaining individual cases in AgeSub (50–54). To explain and demonstrate some key elements in the figure, Figure 6a,b should be the primary focus. First, in Figure 6a, at the top-right corner, $f\left(x\right)=0.356$ is the predicted outcome from the ML model for the positive case, which has a likelihood value between 0 and 1. Because the data in this study have a class imbalance issue, the threshold for determining whether the outcome should be positive or not has been adjusted, for which f(x)≥0.213 is determined as positive in AgeSub (45–49). Second, in Figure 6a, the x-axis represents the SHAP values, and the y-axis represents the features and their corresponding values for an individual case. Third, in the middle section of Figure 6a, the red bar indicates a positive impact on the outcome, whereas the blue bar indicates a negative impact. Fourth, at the bottom of Figure 6a, $E\left[f\left(x\right)\right]=0.198$ is the expected value (EV). The EV, also known as the background data in the SHAP method, is the actual percentage of positive cases from the training data used when building the ML model. The EV represents a naïve predicted outcome and can be considered a starting point. By adding the SHAP values of each feature and EV, the sum is equal to the outcome. In other words, using EV as the starting point and $f\left(x\right)$ as the endpoint, the SHAP values can indicate how each feature contributes to the outcome relatively. Thus, for the case shown in Figure 6a, a breast self-examination of 2 (mass, pain, or tenderness) and an education level of 4 (postgraduate) are the features contributing the most to a positive outcome.

For the negative case in AgeSub (45–49) in Figure 6b, the elements remain the same as in Figure 6a, with the only differences being the EV and the threshold for determining whether the outcome is negative. Because Figure 6b shows a negative case in AgeSub (45–49), the EV equals the actual percentage of the negative cases in the training data. Moreover, the threshold for negative cases is 1−0.213=0.787, for which f(x)≥0.787 is determined as a negative case. The concept mentioned in this section remains the same as that in Figure 6c,d. For the positive case shown in Figure 6c, the threshold for determination was 0.147, whereas the threshold for the negative case shown in Figure 6d was 0.853. In summary, the SHAP can provide helpful information for gaining more insight into the contribution of each feature to individual outcomes.

## 4. Discussion 

The model successfully demonstrated age at menarche, education level, parity, breast self-examination, and BMI as the top five significant risk factors affecting mammography outcomes. Breast self-examination, education level and reproductive lifespan were ranked higher in the younger group, whereas parity and BMI were favored in the older group. The RF model demonstrated the greatest efficacy, with the highest AUC in the analysis of both age subgroups (Table 3 and Table 4). The SHAP value framework was applied to the RF models to provide insight into the decision-making process by revealing the magnitude of each risk factor on the formulation of the final prediction. 

The results indicated that age at menarche had the greatest impact on mammography outcomes in both subgroups. Early age at menarche is a well-established risk factor for breast cancer, with earlier ages conferring a higher risk [11]. This has been extensively documented in the existing literature, attributing this greater risk to the mitotic effect of excess hormone exposure on the differentiation and proliferation of breast tissue [33]. It has been reported that women at an early age at menarche continue to have higher levels of estrogen for several years after puberty, thus increasing their cumulative lifetime estrogen exposure [34]. Research carried out by Ganz et al. proposed that estrogen contributes to breast cancer risk by influencing cell turnover and increasing breast epigenetic age, concluding that earlier age at menarche and higher BMI were associated with higher breast epigenetic age in healthy breast tissue, thus drawing parallels to the increased risk of breast cancer [35]. The reliability of this association was further evidenced by a meta-analysis of 117 epidemiological studies that concluded that the younger the age of menarche, the higher the relative risk of breast cancer [33]. 

The second most important risk factor identified by the model was education level. This is supported by numerous studies, including a meta-analysis of 18 cohort studies with over 10 million women that associated higher levels of education with an increased risk of breast cancer [36]. Many studies have also equated education level with socioeconomic status and found similarly that higher socioeconomic status confers a greater risk of breast cancer [37,38,39]. It is postulated that this correlation is largely due to the differences in known risk factors for breast cancer between educational levels, such as alcohol consumption, hormone replacement therapy, and parity, as well as participation or lack thereof in mammography screening [40].

Parity was identified as the third most important factor in determining mammography outcomes. Previous studies have concluded similarly, with nulliparous women carrying higher risks of breast cancer [41]. Similarly, another prospective study demonstrated that women with four or more pregnancies lasting longer than six months were associated with a lower relative risk of breast cancer of 0.68 [42]. These differences can be attributed to hormonal changes during pregnancy that reduce breast tissue carcinogenesis, as demonstrated in human and animal studies [43]. Many studies have indicated that breastfeeding lowers the risk of breast cancer by reducing estrogen and progesterone [44,45]. Breastfeeding as a predictor should be considered in tandem with parity, given the absence of breastfeeding in nulliparous women.

Our model implicates breast self-examination as the fourth most important risk factor for predicting mammography outcome. Evidence for the benefits of breast self-examination remains controversial. The American Cancer Society no longer recommends self-examination as a screening method for women in the US, whereas the NHS Breast Screening Programme continues to advocate for self-examination for women in the UK. Population studies have shown increased rates of detection for breast cancer in cohorts advised to perform self-examination [46,47]. However, there exists conflicting research that discredits self-examination as an effective screening tool, with intervention groups showing increased rates of unnecessary biopsies and no overall reduction in mortality [48,49]. Our results suggest that breast self-examination is the fourth most important risk factor for predicting mammography outcomes. This provides evidence in support of breast self-examination as a screening tool, given that positive mammography outcomes were defined as results that required further follow-up or intervention.

BMI was the fifth most influential factor identified when averaged across both age groups, which is supported by abundant literature demonstrating obesity as one of the leading modifiable risk factors in the development of breast cancer [50,51]. The mechanistic relationship in which obesity promotes breast cancer can be explained via the estrogenic and inflammatory nature of adipose tissue, subsequently jeopardizing the development of normal breast tissue [52,53]. Although several large studies and meta-analyses have shown a positive association between BMI and breast cancer risk in postmenopausal women [54,55], the link between BMI and breast cancer risk in premenopausal women remains unclear [56,57,58,59]. This phenomenon was supported by our results, which ranked the BMI category higher in the older age group. Given that the mean age of menopause in Taiwan was reported as 50.2 years old in 2020 [60], conclusions from the older age group can be extrapolated and applied to the postmenopausal population. 

Reproductive lifespan was identified as a significant factor in the younger age group. This variable describes the years between age at menarche and age at menopause, during which the ovaries produce hormones that have a direct effect on breast tissue development [11]. A prospective study conducted by Monninkhof et al., including 10,591 women, demonstrated positive associations between earlier menopause and, thus, a shorter reproductive lifespan, and lower subsequent breast cancer risk [61]. This is supported by a recent meta-analysis of over 40,000 women who showed an increased breast cancer risk every year during menopause [33]. The application of SHAP values demonstrates the absolute impact of reproductive lifespan on mammography outcome; the discrepancy in the ranking of reproductive lifespan between the two age groups allows us to conclude that the protective effects of a shorter reproductive lifespan outweigh the deleterious effects of a longer reproductive lifespan when pertaining to breast cancer risk.

The model-agnostic nature of the SHAP methodology provides many classic ML approaches with much needed interpretability and insight into the ‘black box’ phenomenon. SHAP can explain predictions by computing the contributions of individual variables, accounting for local accuracy, missingness, and consistency, to formulate absolute magnitude and directionality of impact in the prediction of the desired outcome. Whilst conclusions from ML models may be extrapolated to wider populations, individual cases often have their own rankings of predictive variables. The directionality of the effect of risk factors on predicting the outcome may also differ for each case, as shown in Figure 6a–d. However, the promising aspects of SHAP do come with limitations. Feature dependency negatively impacts the ability of SHAP to make predictions through permutating feature values as it operates under the assumption that the variables are independent. This can lead to unrealistic predictions and confusion in model interpretability through inappropriate correlations between proxy variables and the desired outcome [20]. Limitations also exist in the development of the ML model, as our model was trained on questionnaire data from a single center. While measures were taken to validate the data quality, all data were self-reported, thus affecting the model’s predictive sensitivity and specificity. ML data balancing techniques were applied to maximize the accuracy parameters; however, these still resulted in lower sensitivity and specificity when compared to other ML models developed and trained on medical imagery [27,28]. 

To further enhance the applicability and accuracy parameters of our model, a larger dataset across multiple centers is necessary to enhance the data quality. While this study focuses on age groups with the highest incidence of breast cancer, future analysis encompassing older age groups would yield significant conclusions, especially pertaining to the post-menopausal population. The retrospective nature of this study makes it prone to selection bias. The prospective validation of the model, possibly in conjunction with mammographic image recognition neural network models, would be meaningful and result in significant clinical implications.

## 5. Conclusions

Through the analysis of 16 risk factors for breast cancer via RF and SHAP value methodology, this study identified age at menarche, education level, parity, breast self-examination, and BMI category as the five most important factors in predicting mammography outcomes, all of which are supported by the existing literature. Stratifying participants into younger and older age groups allowed for the differences in the magnitude of impact of each risk factor accounting for age to be evaluated. The use of the SHAP value provides transparency and interpretability to ML models, which will hopefully aid clinicians in making medical decisions and increase the acceptability of ML integration into healthcare to alleviate the disease burden. This new methodology will also allow clinicians to identify previously undetected interactions between prognostic variables for each individual case, providing new avenues for research and making progress toward the future of individualized medicine.

## Figures and Tables

**Table 1:** Demographic characteristics of participants.

Characteristics	AgeSub (45–49)	AgeSub (50–54)
	**Mean (SD)**	
**Age at menarche**	13.47 (1.54)	13.77 (1.56)
	**N (%)**	
**BMI category**		
0: BMI < 18.5	271 (5%)	171 (3%)
1: 18.5 ≤ BMI < 24	3496 (61%)	3380 (61%)
2: 24 ≤ BMI < 27	1181 (21%)	1235 (22%)
3: 27 ≤ BMI < 30	452 (8%)	520 (9%)
4: 30 ≤ BMI < 35	253 (4%)	234 (4%)
5: BMI ≥ 35	56 (1%)	45 (1%)
**Education level**		
0: Primary school	95 (2%)	251 (4%)
1: Lower secondary school	330 (6%)	581 (10%)
2: Upper secondary school	1861 (33%)	2224 (40%)
3: University	2787 (49%)	2121 (38%)
4: Postgraduate	636 (11%)	408 (7%)
**Reproductive lifespan**		
0: ≤24	63 (1%)	88 (2%)
1: 25–29	302 (5%)	200 (4%)
2: 30–34	3697 (65%)	951 (17%)
3: 35–39	1647 (29%)	3647 (65%)
4: ≥40	-	699 (13%)
**Number of relatives with confirmed breast cancer**		
0: 0	5181 (91%)	4965 (89%)
1: 1	487 (9%)	579 (10%)
2: ≥2	41 (1%)	41 (1%)

**Table 2:** Clinical characteristics of participants.

Characteristics	AgeSub (45–49)	AgeSub (50–54)
	**N (%)**	
**History of major diseases**		
0: No	4864 (85%)	4603 (82%)
1: Benign	714 (13%)	809 (14%)
2: Cancer (other than breast)	131 (2%)	173 (3%)
**Breast self-examination**		
0: Breast self-exam negative	3896 (68%)	4124 (74%)
1: Never breast self-exam	1394 (24%)	1080 (19%)
2: Mass or pain or tenderness	419 (7%)	381 (7%)
**Mammography within 2 years**		
0: No	2289 (40%)	2436 (44%)
1: Yes	3420 (60%)	3149 (56%)
**History of breast surgery**		
0: No	5258 (92%)	5029 (90%)
1: Yes	451 (8%)	556 (10%)
**Age of first childbirth**		
0: <21	362 (6%)	345 (6%)
1: 21–34	3759 (66%)	4047 (42%)
2: ≥35	432 (8%)	291 (5%)
3: No childbirth	1156 (20%)	902 (16%)
**Parity**		
0: 0 time	1156 (20%)	902 (16%)
1: 1 time	1262 (22%)	958 (17%)
2: 2 times	2563 (45%)	2665 (48%)
3: 3 times	641 (11%)	939 (17%)
4: ≥4 times	87 (2%)	121 (2%)
**Breastfeeding**		
0: Nulliparous	1156 (20%)	902 (16%)
1: No	1861 (33%)	2660 (48%)
2: Yes	2692 (47%)	2023 (36%)
**Age of starting hormone replacement therapy**		
0: No	5529 (97%)	5221 (93%)
1: ≥60	-	-
2: 50–59	-	196 (4%)
3: 40–49	143 (3%)	139 (2%)
4: 30–39	26 (<1%)	22 (<1%)
5: <30	11 (<1%)	7 (<1%)
**Duration of hormone replacement therapy use**		
0: No	5529 (97%)	5221 (93%)
1: <5	153 (3%)	299 (5%)
2: ≥5	27 (<1%)	65 (1%)
**Age of starting oral contraceptives**		
0: No	5488 (96%)	5331 (95%)
1: >25	157 (3%)	155 (3%)
2: ≤25	64 (1%)	99 (2%)
**Duration of oral contraceptive use (years)**		
0: No	5488 (96%)	5331 (95%)
1: ≤5	166 (3%)	187 (3%)
2: >5	55 (1%)	67 (1%)
**Y: Mammogram findings**		
0: Negative	4515 (79%)	4720 (85%)
1: Positive	1194 (21%)	865 (15%)

**Table 3:** Modeling results of AgeSub (45–49) and AgeSub (50–54).

Method	Sensitivity	Specificity	AUC
**AgeSub (45–49)**			
**Lasso**	52.30	63.13	59.02
**XGBoost**	51.37	61.56	56.66
**RF**	**54.98**	**64.42**	**61.62**
**AgeSub (50–54)**			
**Lasso**	54.71	**60.17**	58.21
**XGBoost**	56.31	57.55	57.39
**RF**	**66.67**	54.40	**61.78**

**Table 4:** Best RF result of each age subgroup.

AgeSub	Sensitivity	Specificity	AUC
**45–49**	54.98	**64.42**	**61.62**
**50–54**	**66.67**	54.40	61.78

## Data Availability

Data available on request due to privacy/ethical restrictions.
